# Preliminary Observations on Skeletal Muscle Adaptation and Plasticity in Homer 2^-/-^ Mice

**DOI:** 10.3390/metabo11090642

**Published:** 2021-09-19

**Authors:** Paola Lorenzon, Sandra Furlan, Barbara Ravara, Alessandra Bosutti, Gabriele Massaria, Annalisa Bernareggi, Marina Sciancalepore, Gabor Trautmann, Katharina Block, Dieter Blottner, Paul F. Worley, Sandra Zampieri, Michele Salanova, Pompeo Volpe

**Affiliations:** 1 Dipartimento di Scienze della Vita, Università di Trieste, I-34077 Trieste, Italy; plorenzon@units.it (P.L.); bosutti@units.it (A.B.); GABRIELE.MASSARIA@phd.units.it (G.M.); abernareggi@units.it (A.B.); msciancalepore@units.it (M.S.); 2 Istituto di Neuroscienze del Consiglio Nazionale delle Ricerche, Sezione di Padova, I-35121 Padova, Italy; sfurlan@bio.unipd.it; 3 Dipartimento di Scienze Biomediche, Università di Padova, I-35121 Padova, Italy; barbara.ravara@unipd.it (B.R.); sanzamp@unipd.it (S.Z.); 4 Institute for Integrative Neuroanatomy, Neuromuscular System, Center of Space Medicine Berlin (ZWMB), Charité Universitätsmedizin Berlin, D-10115 Berlin, Germany; gabor.trautmann@charite.de (G.T.); katharina.block@charite.de (K.B.); dieter.blottner@charite.de (D.B.); michele.salanova@charite.de (M.S.); 5 Department of Neuroscience, Johns Hopkins University School of Medicine, 725 N Wolfe Street, Baltimore, MD 21205, USA; pworley1@jhmi.edu; 6 Dipartimento di Scienze Chirurgiche, Oncologiche e Gastroenterologiche, Università di Padova, I-35122 Padova, Italy

**Keywords:** Homer 2, atrophy, neuromuscular junction, skeletal muscle

## Abstract

Homer represents a diversified family of scaffold and transduction proteins made up of several isoforms. Here, we present preliminary observations on skeletal muscle adaptation and plasticity in a transgenic model of Homer 2^-/-^ mouse using a multifaceted approach entailing morphometry, quantitative RT-PCR (Reverse Transcription PCR), confocal immunofluorescence, and electrophysiology. Morphometry shows that *Soleus* muscle (SOL), at variance with *Extensor digitorum longus* muscle (EDL) and *Flexor digitorum brevis* muscle (FDB), displays sizable reduction of fibre cross-sectional area compared to the WT counterparts. In SOL of Homer 2^-/-^ mice, quantitative RT-PCR indicated the upregulation of *Atrogin-1* and *Muscle ring finger protein 1 (MuRF1)* genes, and confocal immunofluorescence showed the decrease of neuromuscular junction (NMJ) Homer content. Electrophysiological measurements of isolated FDB fibres from Homer 2^-/-^ mice detected the exclusive presence of the adult ε-nAChR isoform excluding denervation. As for NMJ morphology, data were not conclusive, and further work is needed to ascertain whether the null Homer 2 phenotype induces any endplate remodelling. Within the context of adaptation and plasticity, the present data show that Homer 2 is a co-regulator of the normotrophic *status* in a muscle specific fashion.

## 1. Introduction

Homer proteins are scaffolds and transducers that play a central role in Ca^2+^ signalling, development, and adaptation in skeletal muscle [1,2,3,4,5]. There are three Homer genes, *Homer 1*, *Homer 2*, and *Homer 3,* each encoding for several transcripts [3,6]. Homer 1 and Homer 2 interact with both sarcoplasmic reticulum Ca^2+^ release channels, IP_3_R and RYR1 [7,8], whereas Homer 1b/c also functions as an important scaffold for transient receptor potential (TRP) channels and thus regulates mechanotransduction in skeletal muscle [2]. Homer 1 proteins act as dynamic regulators of RyR1 activity, and the equilibrium between short and long Homer proteins at the receptor site defines the RyR1 channel activity [9]. Homer 1 expression is identical irrespective of the muscle type, whereas expression of Homer 2a/b appears to be characteristic of the slow-twitch phenotype. In general terms, distribution of Homer 2 in skeletal muscles appears to be species independent and fibre type-dependent [1,3].

Homer 1 is either diffusely distributed at the I band with reinforcement of the Z line [10] or localises to the Z-disk [2]; transgenic Homer 1^-/-^ mice [11] exhibit a skeletal myopathy characterised by abnormal TRP channel activity [2]. Homer 2, on the other hand, displays a regular sarcomeric pattern and localises at the Z line level, away from the A-I band where E-C coupling takes place [4].

Homers appear to be key players of skeletal muscle plasticity [5]. In several experimental models of skeletal muscle atrophy, i.e., denervation and disuse (bed rest, hind limb unloading, microgravity), expression of Homer 2 is rapidly and largely inhibited. Homer 2 appears to be required for trophic homeostasis of slow-twitch skeletal muscle fibres [4]. Mechanistically, Homer 2 was shown to antagonise protein degradation in rat slow-twitch skeletal muscles. In particular, downregulation of Homer 2 is an early event of denervation atrophy, i.e., the transcript being reduced by 90% after 3 days. In addition, Homer 2 participates in the control of ubiquitinisation and ensuing proteolysis via transcriptional downregulation of *MuRF1, Muscle Atrophy F-box* (*MAFbx*)*/Atrogin-1*, and *Myogenin* [4].

Homer 2 is observed in the NMJ postsynaptic domain both in slow- and fast-twitch skeletal muscle fibres [12]. Since the Homer 2 isoform is mainly expressed in the slow-twitch fibre type, the role of Homer 2 at NMJ level has been mainly studied in predominantly slow-twitch muscles. In SOL, Homer 2 is downregulated after either denervation or disuse, suggesting a neuronal control mediated by muscle activity. Moreover, at the postsynaptic compartment of NMJ, Homer 2 co-localises and interacts with NFATc1 indicating that Homer 2 translates neuromuscular synaptic input to the calcineurin-NFAT signalling cascade [12].

NMJ stability requires correct expression, proper turnover, and cluster distribution of nicotinic acetylcholine receptors (nAChRs), processes controlled by release of soluble factors from motor nerve endings and electrical activity of muscle fibres [13,14,15]. The interplay among NMJ activity, Ca^2+^ influx through nAChR, and its amplification via Ca^2+^ release from intracellular IP_3_R-sensitive stores is being unveiled [16]. Since IP_3_Rs are enriched at the endplate of skeletal muscle fibres, the current hypothesis is that IP_3_-induced Ca^2+^ release has a major effect on junctional nuclei and synaptic gene expression [17]. At the NMJ level, the role of Homer 2 still is an open issue, and it is not known whether and how it modulates the structure and/or function of endplate. However, Homer 2 might potentially behave as both scaffold and transducer.

Availability of transgenic Homer 2^-/-^ mice [18] allows for further molecular investigation of the trophic role of Homer 2 in skeletal muscle. Moreover, the KO model lends itself as a precious tool to dissect the role of Homer 2 in structure/function of the NMJ.

## 2. Results

### 2.1. SOL, EDL, and FDB Morphometry in WT and Homer 2^-/-^ Mice

An established index of the skeletal muscle trophic state is the fibre cross-sectional area (CSA; [19]), as determined by morphometry in haematoxylin and eosin (H-E)-stained transverse cryosections. Analysis was carried out on transverse sections of SOL and FDB muscles derived from either Homer 2^-/-^ and WT mice [20].

**Figure 1:**
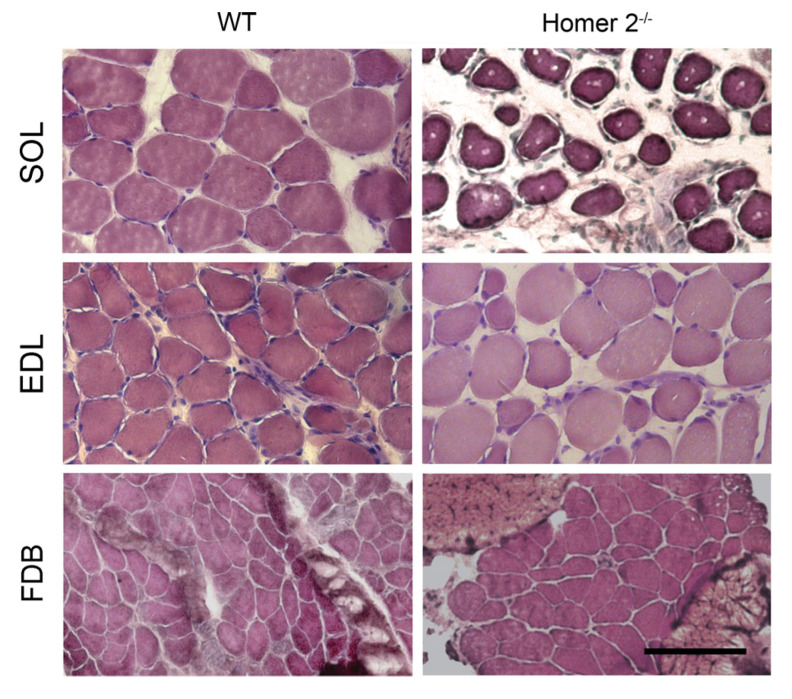
Light microscopy of SOL, EDL, and FDB cryosections from WT and Homer 2^-/-^ mice. Representative haematoxylin and eosin staining of transverse sections of SOL (upper panels), EDL (middle panels), and FDB (lower panels) from WT and Homer 2^-/-^ mice. In EDL of Homer 2^-/-^ mice, some angular fibres were observed. Scale bar = 100 µm.

In SOL muscles from Homer 2^-/-^ mice, mean CSA was decreased by about 24%, as compared to that of WT mice. No significant changes were detected in EDL and FDB muscles (Figure 1 and Table 1). Moreover, SOL from Homer 2^-/-^ mice (Figure 1, right, upper panel) contained smaller fibres interdispersed within a homogeneous matrix.

### 2.2. Quantitative RT-PCR Analysis of MuRF1 and Atrogin-1 Genes in SOL of WT and Homer 2^-/-^ Mice

**Figure 2:**
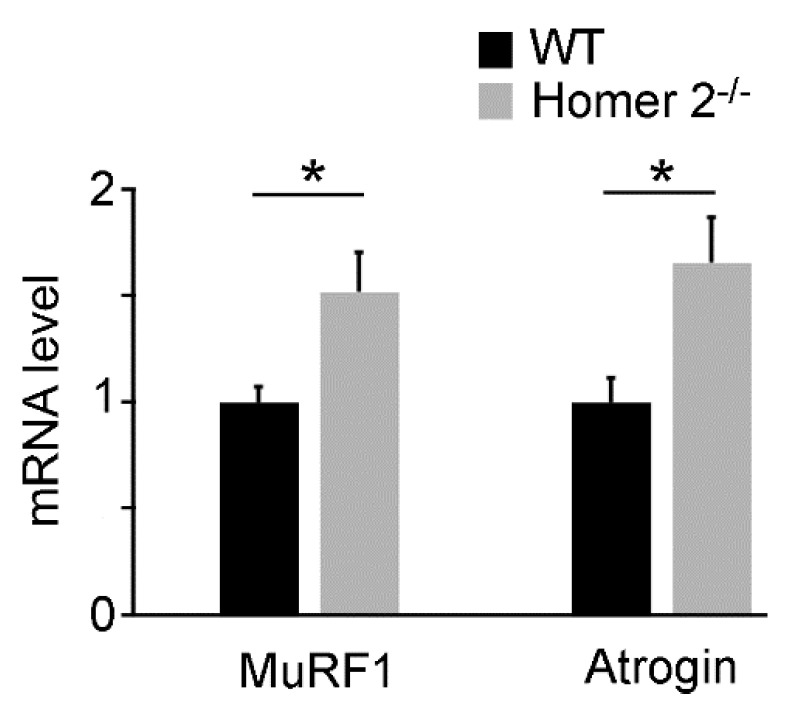
Quantitative RT-PCR analysis of mRNA levels for *MuRF1* and *Atrogin-1* in SOL from WT and Homer 2^-/-^ mice. Values are expressed as the fold change over WT after GeNorm normalisation. Each histogram represents the data obtained from 8 different animals per experimental group. Data are expressed as mean ± SEM. Statistical differences were determined by unpaired *t*-test. * *p* < 0.05 vs. the respective WT control.

Skeletal muscle atrophy upregulates both *MuRF1* and *Atrogin-1* genes [21,22], which encode E3-ubiquitin ligases and are causally involved in denervation atrophy [23]. *Homer 2* replacement, as referred to above, partially counteracted denervation-induced upregulation of both *MuRF1* and *Atrogin-1* genes in rat SOL [4]; thus, hypotrophy/atrophy of SOL from Homer 2^-/-^ mice might be due to the lack of constitutive downregulatory activity exerted by Homer 2. Measurements of *MuRF 1* and *Atrogin-1* transcripts were carried out by quantitative RT-PCR in SOL derived from Homer 2^-/-^ mice. The results show that both *MuRF 1* and *Atrogin-1* genes were significantly upregulated by approximately 35% as compared to the WT muscle (Figure 2).

These data strengthen previous observations on the role of Homer 2 as co-regulator of SOL skeletal muscle normotrophic *status*.

Levels of *Homer 1b/c* and *Homer 1a* mRNA transcripts were also measured in total RNA extracted from SOL of either WT or Homer 2^-/-^ mice. No adaptive changes were detected with respect to the prevalent Homer 1 isoforms expressed in skeletal muscles (data not shown).

### 2.3. Homer Immunoreactivity in SOL from WT and Homer 2^-/-^ Mice

**Figure 3:**
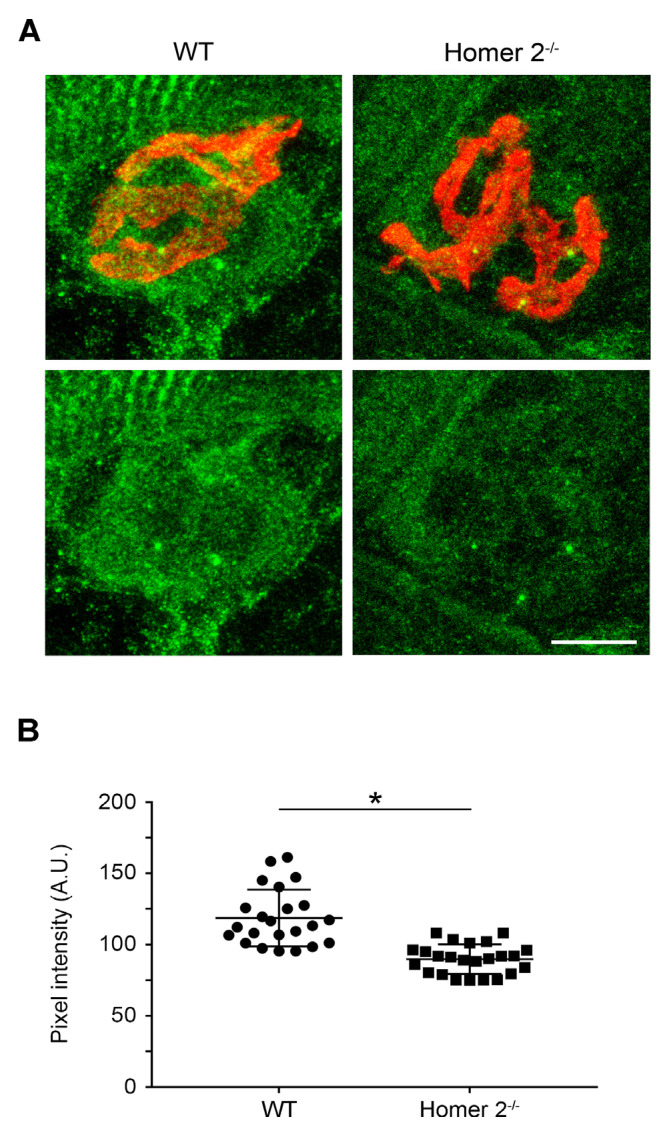
Homer immunoreactivity at the NMJ of SOL cryosections from WT and Homer 2^-/-^ mice. (**A**) SOL cryosections were stained for Homer using a pan-Homer antibody (green) and for nAChRs using Alexa-555-α-BuTX (red) as described in the Materials and Methods section. Confocal microscopy revealed a reduced immunoreactivity for Homer in the NMJ subsynaptic region in Homer 2^-/-^ compared to WT mice (lower panels). Scale bar, 10 µm. (**B**) Data are shown as pixel intensity at the NMJ junction from either WT and Homer 2^-/-^ mice. Data were obtained from 3 animals per each experimental group, and at least 23 endplates per group was analysed. Data are expressed as mean ± SEM. Statistical differences were determined by unpaired *t*-test. * *p* < 0.05 vs. the respective WT control.

Investigation on the presence of Homer was specifically carried out at the NMJ in SOL from WT and Homer 2^-/-^ mice using confocal microscopy. SOL cryosections were double stained with a pan-Homer antibody and Alexa-555-α-BuTX (Figure 3A). Using confocal immunofluorescence microscopy, Homer immunoreactivity resulted decreased by about 25% under the NMJ of Homer 2^-/-^ mice, confirming the localisation of Homer 2 isoform at the subsynaptic level (Figure 3B). The persistence of the other Homer isoforms whose transcription was found to be unchanged (see above) well explains the partial decrease of the immunoreactivity detected by the isoform non-selective pan-Homer antibody.

### 2.4. Endplate Volume in Intact SOL and EDL from WT and Homer 2^-/-^ Mice

**Figure 4:**
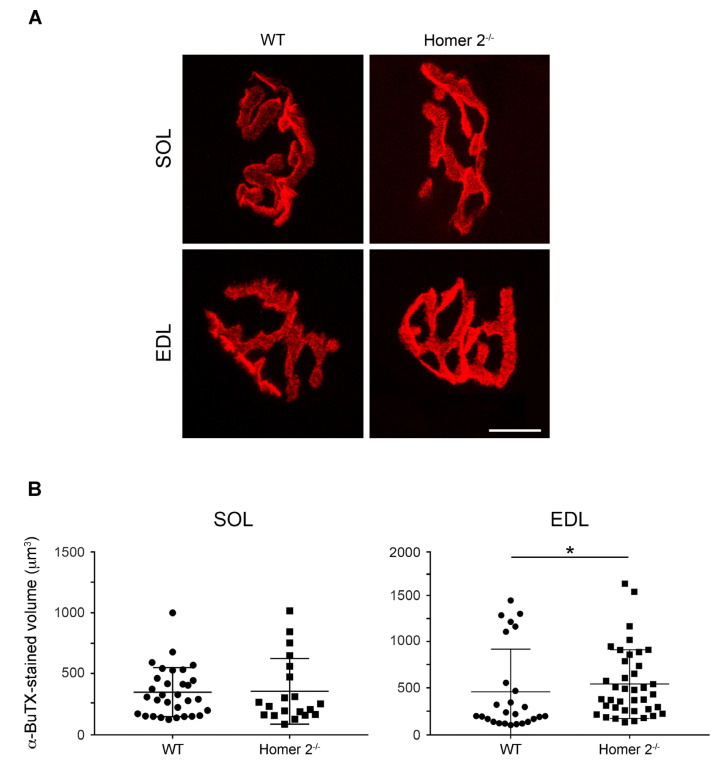
Endplate volume in either SOL and EDL from WT and Homer 2^-/-^ mice. (**A**) SOL and EDL cryosections were stained for nAChRs with Alexa-555-α-BuTX (see the Materials and Methods section for details). Scale bar, 10 µm. (**B**) The endplate volume was calculated both in SOL and EDL as described in the Section 4 [24]. For each group, SOL and EDL from 4 mice were analysed and at least 20 endplates per group were examined. Data are expressed as mean ± SEM. Statistical differences were determined by Mann–Whitney test. * *p* < 0.05 vs. the respective WT control.

Given the reported subsynaptic localisation (see above, and [12]), KO of Homer 2 gene might generate effects at the endplate. Thus, the endplate volume was estimated in intact and frozen SOL and EDL muscles from WT and Homer 2^-/-^ mice. To do this, cryosections were stained with Alexa-555-α-bungarotoxin (α-BuTX) in order to label nAChRs. Under the prevailing experimental conditions and assumptions detailed in the Materials and Methods section, α-BuTX-positive regions show the “pretzel-like” structure characteristic for the mouse endplate (Figure 4A) [24]. The α-BuTX-stained volume did not change in SOL of Homer 2^-/-^ mice (Figure 4B, left hand panel), whereas it was slightly but significantly increased in EDL (Figure 4B, right hand panel).

### 2.5. The Endplate Volume in Single FDB Fibres from WT and Homer 2^-/-^ Mice

**Figure 5:**
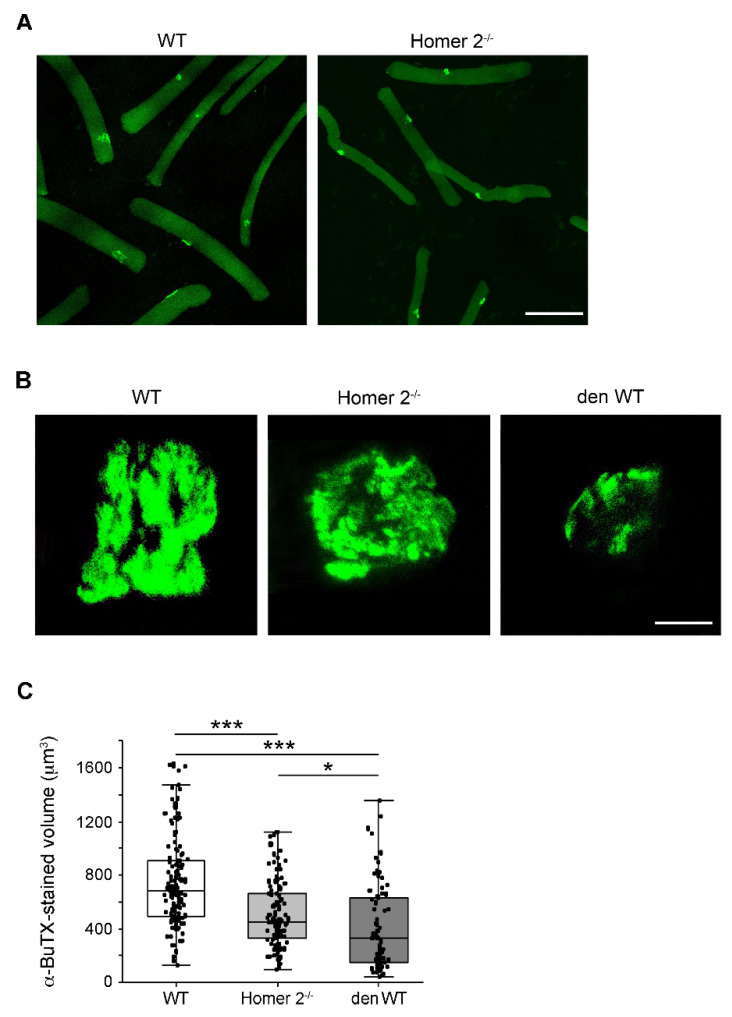
The endplate morphology in WT and Homer 2^-/-^ FDB muscle fibres. (**A**) nAChR staining with Alexa-488-α bungarotoxin (α-BuTX) showed that nAChRs were localised at the endplate level in WT as well as in Homer 2^-/-^ muscle fibres. Scale bar, 150 µm. (**B**) A pretzel-like structure was appreciable in both WT and Homer 2^-/-^ muscle fibres, whereas the endplate appeared more disarrayed in in vitro denervated WT FDB muscle fibres (i.e., 7-days cultured; den WT). Scale bar, 10 µm. (**C**) Volumetric analysis of the α-BuTX staining showed a significant decrease in the volume measured in Homer 2^-/-^ compared to WT (Homer 2^-/-^: 501.86 ± 23.48 µm^3^, *n* = 107 fibres from 5 mice; WT: 752.82 ± 30.13 µm^3^, *n* = 129 fibres from 16 mice). The decrease in volume resulted larger in den WT (404.55 ± 37.10 µm^3^, *n* = 74 fibres from 12 mice) than in Homer 2^-/-^. Data are expressed as mean ± SEM. Data were analysed using one-way ANOVA, and significant differences were analysed by Dunn’s multiple comparisons test; * *p* < 0.05, *** *p* < 0.001.

Isolated FDB skeletal muscle fibres from either WT or Homer 2^-/-^ mice were stained with Alexa-488-α-BuTX to evaluate the endplate volume at single cell level. As in intact EDL and SOL muscles, confocal scanning microscopy detected nAChRs confined to the endplate region (Figure 5A) and organised in a pretzel-like structure in muscle fibres from WT and Homer 2^-/-^ mice (Figure 5B). Analysis of the fluorescent signals reveals that the mean α-BuTX-stained volume was reduced by about 30% in Homer 2^-/-^ FDB skeletal muscle fibres, as compared to WT fibres (see also Figure 5C), formally resembling the effects of in vivo denervation [25,26,27,28]. A set of experiments was carried out in FDB muscle fibres maintained in culture to recapitulate the denervation effects in vitro [29]. In in vitro denervated fibres, endplates displayed more severe alterations, i.e., evident fragmentation and larger reduction in the mean α-BuTX-stained volume (approximately 45%).

### 2.6. Patch Clamp Experiments in Single FDB Fibres from Homer 2^-/-^ Mice

**Figure 6:**
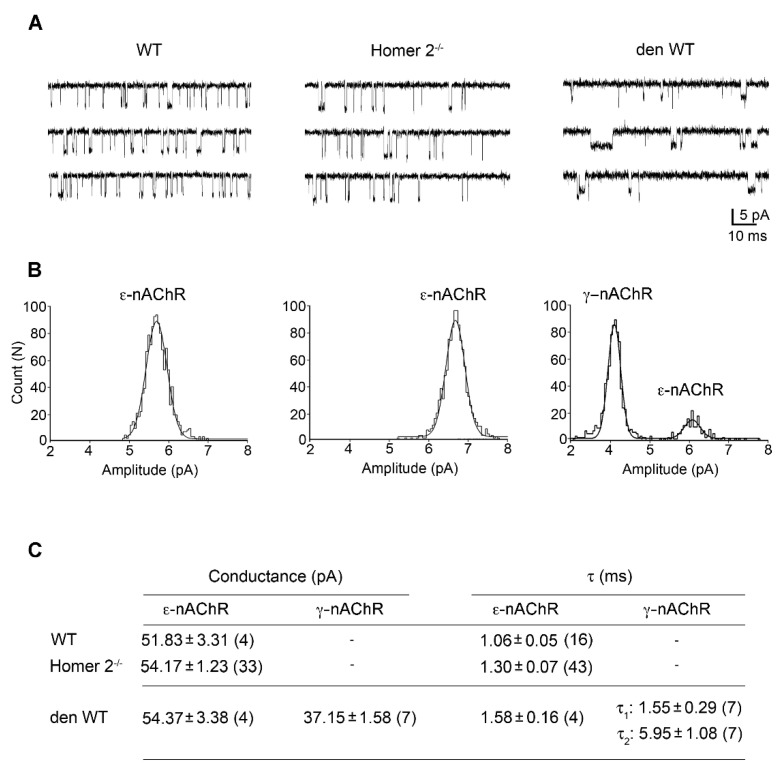
Single-channel activity of nAChRs recorded from FDB endplates. (**A**) Representative single nAChR channel activity evoked by 200 nM ACh at the endplate regions of WT, Homer 2^-/-^, and in vitro denervated WT FDB muscle fibres (i.e., 7-days cultured; den WT). At a pipette potential of +60 mV, channel openings appeared as downward deflections. (**B**) Corresponding unitary current amplitudes with the best fits superimposed. The analysis revealed the presence of the adult ε-nAChR openings alone in WT and Homer 2^-/-^ and the coexistence of adult ε-nAChR and foetal γ-nAChR openings in den FDB muscle fibres. (**C**) Mean values of conductance and time constants of the nAChR openings in WT, Homer 2^-/-^, and den WT muscle fibres. Data are expressed as mean ± SEM. In parentheses, the number of the analysed recordings. Data were from 3 mice for WT, 15 mice from Homer 2^-/-^, and 7 mice for den WT. The statistical analysis performed using one-way ANOVA and Tukey’s post hoc test revealed no statistical differences among groups.

Following FDB dissociation, the exposure of the endplate region makes feasible the electrophysiological recording of the nAChR activity [26,29]. A set of patch clamp experiments was carried out in the cell-attached configuration in order to ascertain which nAChR isoforms are present at the endplate region of Homer 2^-/-^ FDB muscle fibres. ACh (200 nM) was used in the recording pipette solution. The activity of single nAChR channels was measured in membrane patches at the level of the endplate region (Figure 6A), identifiable by phase-contrast microscopy as a roughness of the cell surface [30]. The unitary current amplitude distribution was best fitted by a single Gaussian curve and the open-time distribution by a single exponential, revealing the presence of a single type of nAChR channel (Figure 6B). Values of mean single-channel conductance and time constant confirmed the exclusive presence of the adult ε-nAChR isoform in Homer 2^-/-^ muscle fibres, as in the case of WT muscle fibres (Figure 6C; see also [27]). This is in contrast with the in vitro denervated WT counterpart in which the embryonic γ-nAChR isoform was also expressed (Figure 6C; see also [27]), as it occurs in vivo for denervated skeletal muscle [26].

## 3. Discussion

Changes in skeletal muscle mass may result from either changes in protein turnover, reflecting the balance between protein synthesis and protein degradation, or changes in cell turnover, reflecting the balance between myonuclear accretion and myonuclear loss [31].

Skeletal muscle atrophy occurs when protein degradation rates exceed protein synthesis and takes place in a variety of conditions, including starvation, disuse, denervation, cancer cachexia, and aging. Two major protein degradation pathways, the proteasomal and the autophagic-lysosomal pathways, are involved during muscle atrophy and variably contribute to the loss of muscle mass. Moreover, satellite cells and myonuclei may undergo apoptosis during muscle atrophy [31,32].

Skeletal muscle fibre types are differentially sensitive to specific pathophysiologic atrophy signals. For example, red, slow-twitch oxidative fibres have a higher rate of protein synthesis and degradation and are more resistant to fasting than white, fast-twitch glycolytic fibres. In contrast, slow-twitch fibres are more sensitive to inactivity, microgravity, and denervation-induced atrophy [33].

Our observations indicate that SOL muscle has adapted to genetic lack of *Homer 2*. The *Homer 2* null phenotype is characterised by upregulation of the atrophy master genes *Murf1* and *Atrogin-1*, and 2-month-old mice display clear-cut atrophy. Thus, there appears to be a solid link between either downregulation or absence of *Homer 2* and initiation of muscle catabolism. If Homer 2 contributes to the normotrophic *status* of SOL muscle, genetic lack of *Homer 2* might very well determine hypotrophy rather than atrophy.

In a previous report [4], an almost complete disappearance of Homer 2 was detected after 14-day denervation of rat SOL muscle in parallel with the decrease of both muscle mass and fibre CSA, indicating that the extent of muscle atrophy caused by denervation was likewise conspicuous. Moreover, *Homer 2* replacement by transient transfection of denervated SOL muscle with plasmidic-Homer 2 cDNA was able to partially reverse fibre CSA decrease by about 20–30% [4]. On the basis of these findings, SOL of Homer 2^-/-^ mice was expected to be atrophic for congenital lack of *Homer 2*. The extent of hypotrophy/atrophy observed in Homer 2^-/-^ mice was smaller than that induced by denervation. This observation suggests that the normotrophic *status* of SOL muscle is dependent upon constitutive Homer 2 expression in combination with additional, independent factors linked to the innervated condition of the muscle.

In SOL muscle of Homer 2^-/-^ mice, the decrease of CSA was accompanied by an apparent thickening of the extracellular matrix. At steady state, in 2-month-old Homer 2^-/-^ mice, is this due to either necrosis, apoptosis, lack of satellite cell proliferation, or a combination thereof? Apoptosis is a hallmark of atrophy [31,32] and might explain at least in part such a reduction. Fibrous proliferation would imply a certain degree of preceding muscle necrosis, regeneration, and exhaustion of regeneration. Interference of satellite cell proliferation by Homer 2 might be postulated on the account of our own previous findings on rat SOL regeneration, showing that expression levels of *Homer 2* were positively and linearly related to muscle mass increase during regeneration: *Homer 2* is expressed by myogenic cells during regeneration and increase of *Homer 2b* during regeneration is associated to muscle mass recovery [3]. Moreover, *Homer 2* mRNA and protein were found to be transiently expressed at high levels in whole embryo at developmental stage E14.5 [1]; lack of such a spike might alter the overall skeletal muscle developmental program.

Another interesting finding was that in Homer 2^-/-^ EDL and FDB muscles, no significant changes in CSA were detected. Assessment of different Homer 2 controlled routes in different muscles awaits additional experimental work and could contribute to the identification of putative fibre type-specific signalling pathways controlling atrophy.

Because of their unique molecular properties that allow for clustering and functional modulation of different interacting partners, Homer proteins, and in particular Homer 2 at the subsynaptic domain, could play an important role at the NMJ integrating different downstream signalling pathways controlled by as yet unknown presynaptic signalling mechanisms. Two relevant biological questions are in order: (1) Is there a relationship between NMJ activity, or even NMJ disruption, and initiation of muscle catabolism mediated by Homer 2? (2) Is there a relationship between NMJ activity and muscle development and differentiation mediated by Homer 2?

The present results indicate that the null *Homer 2* phenotype did not entail changes in the nAChRs isoform localisation and expression. Adaptation in Homer 2^-/-^ does not perturb the expression of the adult ε-nAChR isoform, which remains detectable only at endplate level. However, downstream effects in the nAChR signalling pathway cannot be fully excluded.

On the contrary, data pertaining to morphological changes of endplate in Homer 2^-/-^ mice appeared not yet conclusive and conflicting. The apparent discrepancy between data from tissue specimens (Figure 4) and isolated FDB muscle fibres (Figure 5) might be due to at least four different reasons: (1) muscle type and lack of direct identification of fibre type; (2) experimental milieu, frozen muscles vs. isolated and cultured fibres; (3) innervation status (muscle specimens) vs. lack of innervation (isolated fibres); (4) methodology of volumetric analysis. Further work is needed to reconcile the conflicting result, but it is entirely possible, although speculative, that endplate in Homer 2^-/-^ muscle fibres might be more vulnerable to the lack of motor innervation than the WT counterpart.

Since 2005, data in the literature indicate the pivotal role of Homer 2 in muscle differentiation, trophism, and plasticity [1,2,3,4,5,11,12,34]. The present data are in line with the increasing evidence that Homer 2 regulates a few, key transcriptional programs of atrophy. However, more experimental work is needed to better characterise the signalling pathways activated in both slow- and fast-twitch fibres and the integrative function of Homer 2, if any, in the NMJ activity-driven control of skeletal muscle trophism.

## 4. Materials and Methods

### 4.1. Mice and Transgenic Mice

KO mice for *Homer 2* [18] have been generated as described and generously provided by Paul F. Worley (Johns Hopkins School of Medicine, Baltimore, MD, USA); C57BL/6 mice were used to produce *Homer 2* heterozygotes that were crossed to generate WT and Homer 2^-/-^ mice. Even though the WT littermates could show phenotypic properties different from the commercially available C57BL/6 mice, this procedure is commonly used to obtain mice with a more similar genotype except for the gene of interest. All mice used in the present study were bred and housed in the certified animal facility at the University of Trieste. Mice had free access to food and water and were maintained in a 12 h light/12 h dark cycle. Mice between 6 and 9 weeks of age were used for the experiments; body weight of WT and Homer 2^-/-^ mice was determined, and no difference was detected between the two experimental groups. For muscle dissection, mice were sacrificed by cervical dislocation as approved by the local Animal Care Committee and in agreement with the European legislation (2010/63/EU).

### 4.2. Morphometry

The hindlimb EDL, SOL, and FDB muscles were excised from WT and Homer 2^-/-^ mice, placed in OCT, and quickly frozen in liquid nitrogen. Cryosections from any muscle were stained with haematoxylin and eosin (Sigma-Aldrich, Darmstadt, Germany). Morphometry was carried out using ImageJ software (ver 1.42 q, National Institute of Health, Bethesda, MD, USA). Images at magnification 10× were acquired using a Zeiss AxioSkop microscope connected to a Leica DC 300F camera. Cross-sectional area (CSA) of the muscle fibres (µm^2^) was calculated from cross-sections. CSA was estimated by outlining the profile on the monitor screen using a computer mouse.

### 4.3. RNA Extraction and Quantitative RT-PCR

Frozen tissue samples were ground to a fine powder under liquid nitrogen, and total RNA was extracted using Trizol method, following the manufacturer’s instructions and including a glycogen co-precipitating step. Reverse transcription was performed on 1 μg of total RNA by using a SuperScript VILO cDNA Synthesis Kit (ThermoFisher Scientific, Waltham, Massachusetts, MA, USA).

Specific primers for Homer1A and Homer1B/C were designed with the Primer3 software (Whitehead Institute for Biomedical Research, Cambridge, MA, USA; http://frodo.wi.mit.edu/ (accessed on 15 January 2021)), and their thermodynamic specificity was determined using BLAST sequence alignments (U.S. National Center for Biotechnology Information (NCBI), Bethesda, MD, USA) and Vector NTI^®^ Software (Thermo Fisher Scientific, Waltham, Massachusetts, MA, USA). Primers sequences for *MuRF1*, *Atrogin-1*, *Cyclophilin A* (*PPIA*), *TATA-box-binding protein (TBP1*), and *hypoxanthine-guanine phosphoribosyltransferase* (*HPRT1*) were already published in [34]. Quantitative RT-PCR was performed in triplicate in a CFX96 Thermal Cycler (Bio-Rad, Hercules, CA, USA) using SYBR Green chemistry. A melt-curve analysis was performed at the end of each experiment to verify that a single product per primer pair was amplified. *PPIA*, *TBP*, and *HPRT1* were used as reference genes, and normalisation was performed using GeNorm software (V3.5, 2007, https://genorm.cmgg.be (accessed on 15 January 2021)).

Primers sequences were as follows:
*MuRF-1* (Trim63)Fw 5′-ACCTGCTGGTGGAAAACATC-3′
Rv 5′-CTTCGTGTTCCTTGCACATC-3′*Atrogin-1* (Fbxo32)Fw 5′-GCAAACACTGCCACATTCTCTC-3′
Rv 5′-CTTGAGGGGAAAGTGAGACG-3′*Homer 1a*Fw 5′-GAAGTCGCAGGAGAAGATGG-3′
Rv 5′-GAACTTCCATATTTATCCA-3′*Homer 1b/c*Fw 5′- GAAGTCGCAGGAGAAGATGG-3′
Rv 5′-TAGCTCAGCCTCCCAGTGTT-3′*PPIA*Fw 5′-AGCATGTGGTCTTTGGGAAGGTG-3′
Rv: 5′-CTTCTTGCTGGTCTTGCCATTCC-3′*TBP1*Fw: 5′-TCAAACCCAGAATTGTTCTCC-3′
Rv: 5′-AACTATGTGGTCTTCCTGAATCC-3′*HPRT1*Fw: 5′-CTCATGGACTGATTATGGACAGGAC-3′
Rv: 5′-GCAGGTCAGCAAAGAACTTATAGCC-3′

### 4.4. Staining and Image Analysis of SOL and EDL Muscles

EDL and SOL were isolated from WT and Homer 2^-/-^ mice, quickly frozen in liquid nitrogen, and then cut to 10 µm and 30 µm thick sections at the middle portion of the muscle belly, then fixed with 4% (*w*/*v*) paraformaldehyde at RT for 10 min.

For the analysis of the endplate volume, after permeabilisation with a blocking buffer (0.3% Triton, 2% goat serum, and 2% Albumin in TBS, pH 7.4) for 60 min at RT, the 30 µm thick sections were incubated at 4 °C for 48 h with anti-Dystrophin primary antibody (0.076mg/mL in blocking buffer; rabbit Abcam, Cambridge, UK), followed by a 48 h incubation at 4 °C with Alexa-488-secondary antibody (0.2 µg/mL; goat-anti-rabbit ThermoFisher Scientific). At the end, the sections were incubated with Alexa-555-α-BuTX (1µg/mL, ThermoFisher Scientific) for 90 min at RT.

Immunostaining for Homer was carried out using 10 µm thick sections by incubation for 24 h at 4 °C with the primary rabbit anti-Homer antibody (propriety of Dr. Michele Salanova, 0.05 mg/mL), followed by an incubation with the same Alexa-488-goat-anti-rabbit secondary antibody used for the 30 µm thick sections.

Labelled 10 µm and 30 µm thick sections were mounted with a non-curing Mounting medium (ThermoFisher Scientific) and stored at 4 °C for further imaging. Cell nuclei were counterstained by 4′,6-diamidino-2-phenylindole (DAPI, 1:50; Sigma-Aldrich). Labelled sections were imaged using an SP8 confocal laser-scanning microscope (Leica Biosystems, Wetzlar, Germany). Image acquisition was carried out with the 63X/1.3 (NA) using an oil-immersion objective. Optical images were collected at 0.44 μm z resolution by sequential line scanning. Regions of interest (ROIs) were defined as α-BuTX-positive regions that resembled the “pretzel-like” morphology. ROIs were scanned at the *z*-axis throughout the whole thickness of the stained tissue. 3D image reconstruction and image analysis were carried out with the Leica 3D Analysis Tool, allowing us to measure the NMJ volume and α-BuTX-signal intensity. Homer signal intensity was measured in the region 5–10 µm below the α-BuTX-positive signal, between the subsynaptic nuclei and the nAChRs.

### 4.5. Isolation of Muscles Fibres from FDB Muscles

Freshly isolated muscle fibres were obtained from the dissociation of FDB muscles of 6 to 8-weeks old Homer 2^-/-^ and WT male mice. FDB muscle fibres were isolated from both hindlimb foot muscles of a single mouse for each preparation. Briefly, immediately after the isolation, FDB muscles were enzymatically treated for 1 h in ice and 1 h at 37 °C with Type I collagenase 0.3% (*w*/*v*; Sigma-Aldrich), in Tyrode’s solution containing (in mM): NaCl 137, KCl 2.7, MgCl_2_ 1, CaCl_2_ 1.8, Na_2_HPO_4_ 0.35, NaHCO_3_ 12, HEPES 25.2, d-glucose 5.5, pH 7.4 NaOH plus 100 U/mL penicillin, 100 µg/mL streptomycin, and 10% foetal bovine serum (Gibco, Burlington, ON, Canada). Single fibres (750–900 for each mouse) were isolated by mechanical dissociation with Pasteur pipettes with decreasing tip diameters and allowed to settle on Matrigel-coated (1 mg/mL; Corning, Tewksbury, MA, USA) glass coverslips accommodated in 35-mm Petri dishes. Cultures were maintained in the physiological saline as above at 37 °C in a humid air atmosphere containing 5% CO_2_. According to the experimental purpose, denervation effects on the endplate were induced in vitro [29] by culturing isolated FDB muscle fibres for 7 days in a medium composed by DMEM high glucose enriched (Sigma-Aldrich) supplemented with horse serum (5%; Gibco), L-glutamine (2 mM), penicillin (100 IU/mL), and streptomycin (100 μg/mL), at 37 °C in a humid air atmosphere containing 5% CO_2_. In these experimental conditions, a disarrangement of the endplate and the appearance of the foetal γ-nAChR isoform occurred as after in vivo denervation [25,26,27].

### 4.6. nAChRs Staining and Image Analysis of Isolated FDB Fibres

nAChRs staining was conducted in FDB muscle fibres 24 h or 7 days after dissociation according to the experimental aims. Cells were fixed with a solution of 4% (*w*/*v*) paraformaldehyde in PBS for 15 min at 4 °C. Fibres were incubated in a blocking solution containing PBS plus 2% bovine serum albumin (BSA; Sigma-Aldrich) for 30 min. nAChRs were labelled by Alexa-488-α-BuTX (ThermoFisher Scientific; 2.5 µg/mL in PBS supplemented with 0.1% BSA, for 1 h at RT). Cells were mounted on microscope slides.

Distribution of nAChRs and morphology of the endplates were analysed on images acquired by a Nikon C1 confocal microscope equipped with an argon laser (457, 477, 488, and 514 nm lines), a 561 nm and a 640 nm diode laser, and a Plan-Apochromat 60X/1.4 (NA) oil-immersion objective. Optical images were collected at either 0.30 or 0.35 μm z resolution by sequential line scanning.

Volumetric endplate analysis was carried out applying Fiji ImageJ software (ver 2.1.0/1.53c, National Institute of Health, Bethesda, MD, USA), quantifying the α-BuTX-stained volume. For each endplate, a stack of images was collected through the entire depth containing the α-BuTX visible signal. The region of interest (ROI) for the measurement of the endplate volume was set by projecting through the *z*-axis planes the most intense pixels of the α-BuTX signal. To each stack of images, a threshold was applied to eliminate the fluorescent noise and the background signal subtracted. The α-BuTX-stained volume was calculated as the sum of α-BuTX-stained volume of each z-stack, taking in account the step size of acquisition.

### 4.7. nAChRs Single Channel Recordings in Isolated FDB Fibres

Single-nAChR recordings were carried out by patch clamp technique in the cell-attached configuration on the endplate region identifiable by phase contrast microscopy as a distinct roughness of the fibre surface [27]. According to the experimental aim, electrophysiological recordings were performed on freshly isolated Homer 2^-/-^ and WT muscle fibres and on WT muscle fibres after 7 days of culturing (in vitro denervated). Single-channel recordings were performed using an Axopatch 200 amplifier (Molecular Devices, Union City, CA, USA) after achieving a giga seal and when the baseline was stable. Signals were sampled at 50 kHz, filtered at 2 kHz with a low pass Bessel filter, and analysed by the pCLAMP 8.0 software package (Molecular Devices, Union City, CA, USA), using a threshold crossing criterion. All records were performed in the presence of 200 nM of ACh (Sigma-Aldrich), and for each stable patch, more than 500 events were analysed [30]. The open time distribution was best fitted with one or more exponentials if appropriate by maximum likelihood method. Lifetimes of the channel openings were expressed as time constants (τ) of the exponential curves. Single-channel conductance was estimated from the slope of the regression line obtained by plotting the current amplitude against the pipette potentials (Vp) at +60, +80 mV, and +100 mV.

## Figures and Tables

**Table 1:** SOL, EDL, and FDB morphometry in WT and Homer 2^-/-^ mice.

Muscle	WT	Homer 2^-/-^	Δ	*p*
SOL CSA (µm^2^)	1779.00 ± 30.98	1353.00 ± 23.92	−23.92%	<0.0001
	(924)	(622)		
EDL CSA (µm^2^)	1681.44 ± 31.22	1722.00 ± 40.63	+2.41%	0.4162
	(829)	(745)		
FDB CSA (µm^2^)	400.00 ± 9.57	414.50 ± 8.51	+7.42%	0.2606
	(670)	(950)		

Data are expressed as mean ± SEM. In parentheses, number of analysed fibres for each experimental group. Statistical differences were determined by unpaired *t*-test. Data were from 3 animals for both WT and Homer 2^-/-^.

## Data Availability

Data are contained within the article.
